# Understanding Patient and Caregiver Attitudes Towards Cognitive and Behavioural Screening in Amyotrophic Lateral Sclerosis

**DOI:** 10.1002/alz70858_105464

**Published:** 2025-12-26

**Authors:** Leslie Ing, Alys Griffiths, Daniel J. Blackburn, Christopher J McDermott

**Affiliations:** ^1^ University of Sheffield, Sheffield, South Yorkshire, United Kingdom

## Abstract

**Background:**

Amyotrophic Lateral Sclerosis (ALS) is a fatal neurodegenerative disease characterised by progressive motor disability. Cognitive and behavioural impairment is increasingly recognised, affecting up to 50% of patients, with 15% developing Frontotemporal Dementia (FTD). While cognitive screening tools like the Edinburgh Cognitive and Behavioural ALS Screen (ECAS) exist, they are not routinely implemented in ALS care. Patient and caregiver perspectives on cognitive testing remain underexplored, limiting understanding of how best to facilitate acceptance and integration into clinical pathways. This study explores attitudes toward cognitive screening in ALS, identifying barriers, facilitators, and perceived impacts to inform patient‐centred approaches.

**Method:**

Semi‐structured interviews were conducted with ALS patients (*n* = 10) and caregivers (*n* = 9) recruited from Sheffield Teaching Hospitals and the UK Motor Neuron Disease Association network. Participants represented a range of disease stages and cognitive‐behavioural symptom severity. Interviews were conducted separately by three researchers. Reflexive Thematic Analysis was used, with iterative coding refinement to develop and finalise an interpretative framework capturing diverse perspectives.

**Result:**

Perceptions of cognitive testing in ALS were highly individual and context‐dependent. While some participants saw early screening as a means of preparedness, others feared it would threaten autonomy. Emotional responses varied, with individuals balancing denial, fear, and acceptance. Caregivers often advocated for screening to aid future planning but faced tensions in respecting patient autonomy. Misattributions of cognitive and behavioural symptoms created additional strain. Practical barriers, including travel, fatigue, and accessibility, further influenced decision‐making. Clinician communication played a crucial role. Clear, empathetic discussions facilitated engagement, whereas overwhelming or unclear information led to hesitancy. Testing was valued when it provided actionable insights for care planning, but concerns arose when results lacked practical application.

**Conclusion:**

Cognitive screening in ALS requires a patient‐centred approach that considers emotional readiness, individual preferences, and logistical challenges. Adaptive clinical strategies were recommended, which personalise communication, offer flexible testing delivery, and ensure results lead to meaningful, actionable outcomes. Findings support the development of remote cognitive screening tools that maintain clinician involvement while reducing testing burden. This could improve acceptance and accessibility of screening, facilitating timely support for those affected.

